# Evaluation of the effects of school zone signs and markings on speed reduction: a driving simulator study

**DOI:** 10.1186/s40064-016-2396-x

**Published:** 2016-06-21

**Authors:** Xiaohua Zhao, Jiahui Li, Jianming Ma, Jian Rong

**Affiliations:** Beijing Engineering Research Center of Urban Transport Operation Guarantee, Beijing Collaborative Innovation Center for Metropolitan Transportation, Beijing University of Technology, Beijing, 100124 China; Road Safety Research Center, Research Institute of Highway Ministry of Transport, Beijing, 100088 China; Traffic Operations Division, Texas Department of Transportation, Austin, TX 78701-2483 USA

**Keywords:** School zone signs and markings, Driving simulator experiment, Driver behavior, Speed, Relatively best alternative design

## Abstract

Traffic control devices are one of the most significant factors affecting driving behavior. In China, there is a lack of installation guidelines or standards for traffic control devices in school zones. In addition, little research has been done to examine the effects of traffic control devices on driving behavior. Few guidelines have been established for implementing traffic control devices in school zones in China. This research conducted a driving simulator experiment to assess the effects of school zone signs and markings for two different types of schools. The efficiency of these traffic control devices was evaluated using four variables derived from the driving simulation, including average speed, relative speed difference, standard deviation of acceleration, and 85th percentile speed. Results showed that traffic control devices such as the Flashing Beacon and School Crossing Ahead Warning Assembly, the Reduce Speed and School Crossing Warning Assembly, and the School Crossing Ahead Pavement Markings were recommended for school zones adjacent to a major multilane roadway, which is characterized by a median strip, high traffic volume, high-speed traffic and the presence of pedestrian crossing signals. The School Crossing Ahead Pavement Markings were recommended for school zones on a minor two-lane roadway, which is characterized by low traffic volume, low speed, and no pedestrian crossing signals.

## Background

As is known to all, it is particularly important for drivers to comply with posted speed limits in school zones. However, speeding-related crashes in school zones have remained unchanged in recent years despite comprehensive national campaigns to promote traffic safety in school zones. A report on national incident statistics indicated that in 2006, there were 110 reported incidents (including food safety incidents, traffic crashes and other incidents) involving primary and secondary school students in China. A total of 188 students died and 1266 were injured in those 110 incidents. Among these incidents, traffic fatalities accounted for 30.26 % of deaths and ranked second according to the statistics from the Ministry of Public Security of China (Liu and Zhang 2008). In the first half of 2013, there were 1267 traffic accidents in Shanghai, China, a total of 1272 students were died or injured (Wang 2014). The primary and secondary school students are more than 30 % among all casualties in traffic accidents each year (Zhang 2014). Therefore, improving traffic safety in school zones is an immediate imperative.

The proper use of traffic control devices is one of the most common and vital ways to improve school zone safety. School zones should be marked with special signage to alert drivers of the high concentration of children. Thus, school crossing signs, speed limit signs, school zone pavement markings and other treatments should be regularly implemented to remind drivers to take special care and attention when driving in school zones (FHWA 2009).

Guidelines and implementation methods for traffic control devices in school zones do exist, but these rules vary from country to country. In the US, Part 7 of the Manual on Uniform Traffic Control Devices (MUTCD) states the principles and standards for controlling traffic in school zones. These principles and standards also provide information on the appropriate design, application, and maintenance of various traffic control devices (including adult school crossing guards, student patrols and grade-separated crossings, school area speed limits and signage and so on).

In China, *Road Traffic Signs and Markings* Manual (GB5768-2009), which is the National Standards of the People’s Republic of China (2009), contains guidelines for implementing traffic control devices in school zones. However, the guidelines are relatively vague, only mention speed limit signs, School Bus Stop Ahead signs, and the School Crossing Ahead Warning Assembly. Besides, it doesn’t offer specific requirements for the of these traffic control devices under various traffic, road and environmental conditions. Therefore, the national standard of the school zone fails to provide sufficient guidance for the proper use of traffic control devices.

Apart from the principles and standards, numerous research studies about traffic control devices in school zones have been conducted in other countries. For example, Rose et al. (2004) analyzed the speed data before and after rear-facing school speed limit beacons were installed in the field, concluding that a flashing beacon at the end of the school zone is a potentially effective means of improving drivers’ compliance with school zone speed limits. The percent of vehicles exceeding 35 mph was significantly reduced by approximately 25–30 %. In addition, Saibel et al. (1999), Hawkins (1993, 2007) and Lindenmann (2005) found that school zone signs with flashing beacons were more effective in slowing traffic than those without flashing beacons. Moreover, Ullman and Rose (2005) and Lee et al. (2006) found that speed monitoring devices (SMD) also had a positive safety impact by reducing traffic speed. The short-term study results showed that about 17.5 % (8.2 km/h) of average speed was reduced at the SMD location. The long-term study results showed that about 12.4 % (5.8 km/h) of average speed was reduced at the SMD location. A study conducted by the Washington, DC Department of Transportation showed that driver feedback signs (DFS) can reduce mean speeds significantly during active school periods (KLS Engineering 2006). Brewer and Fitzpatrick (2009) investigated speed-distance profiles in school zones and found that operating speeds increased as the distance to the beginning of the school zone increased. Tay (2009) found that mean speed (2 km/h) and non-compliance rates (13.5 %) were lower in school zones with chain-link fencing than without fencing.

Until now, most research studies in China have concentrated on determining the causes of traffic crashes. Little research has been devoted to the proper use of traffic control devices and how these traffic control devices affect driving behavior. Liu and Zhang (2008) conducted a questionnaire survey to investigate the causes of traffic crashes in school zones. They found that adverse road conditions and the absence of traffic signs were contributing factors to crashes. Using a field study, Cheng et al. (2011) asserted that a lack of appropriate traffic control devices was one of the most important causes of traffic crashes in school areas.

Previous studies showed that speed is considered as a major factor in fatalities and risk of injury (Kattan et al. 2011). The faster a vehicle is traveling upon striking a pedestrian, the more severe and potentially fatal the injuries will be (Garder 2004). Anderson et al. (1997) suggested that a fatal pedestrian accident was six times less likely to happen if the vehicle’s impact speed was 37 km/h (10 % chance of fatality) as opposed to 45 km/h (60 % chance of fatality). Hence, the Canadian province of Alberta has set the speed limit in school and playground zones at 30 km/h. Therefore, speed is one of the major factors considered in this research. In order to evaluate the decelerating effect of different school zone signs and markings for different types of schools, a driving simulator experiment was employed and the effects of target devices are analyzed and compared.

## Methods

### Driver survey and program design

A field survey was conducted to identify the traffic information needs of drivers. A total of 505 drivers were surveyed, including parents of students who were familiar with the school’s surroundings and other licensed drivers who were unfamiliar with the school’s surroundings.

According to the results of the field investigation, there were 18 kinds of signs and markings regularly used in school zones. Table 1 summarizes the major results and findings of the survey. Six kinds of signs and markings were favored by more than half of the respondents, indicating that they considered these to be the most effective devices. Therefore, these six devices were chosen as the focus in this study.

**Table 1:** Summary of responses for driver information survey

Abbreviation	Signs and markings	Meaning of the sign and marking	Percentage of approval by drivers
WA		School Crossing Ahead Warning Assembly	82
IS		School Crossing Ahead Informational Sign	67
SL		Speed limit sign	65
RSWA		Reduce Speed and School Crossing Warning Assembly	62
PM		School Crossing Ahead Pavement Markings	58
FBWA		Flashing Beacon and School Crossing Ahead Warning Assembly	53

**Fig. 1:**
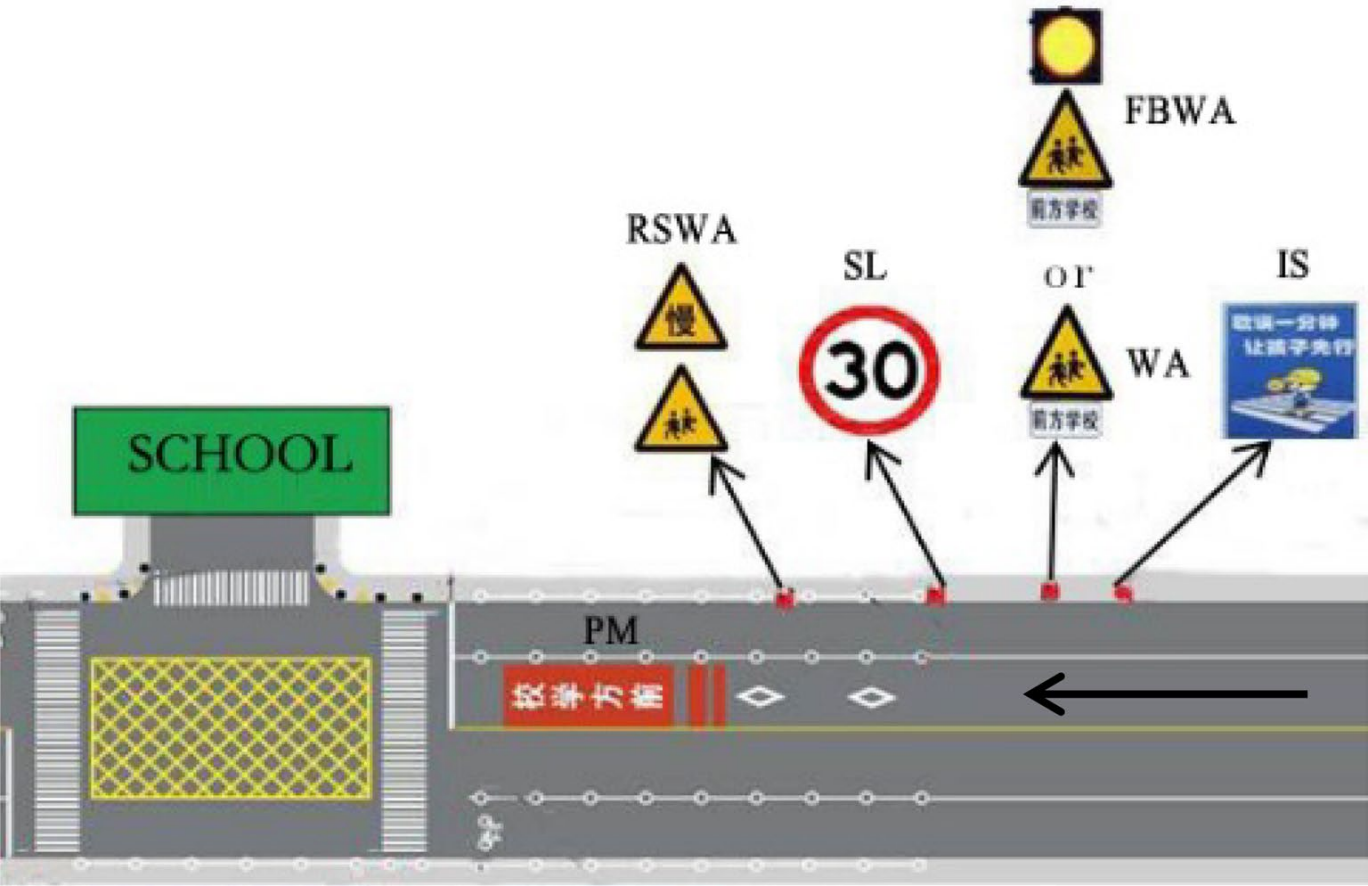
The placement for six kinds of signs and markings

It is worth mentioning that the speed limit sign’s effectiveness in reducing speeds in school zones is mixed. The findings may differ from one country to another. For example, Lindenmann (2005) found that collision frequency and severity declined significantly in the school zone after the installation of 30 km/h speed limit signs. However, Young and Dixon (2003) found that school zone signage had no significant effect on speed. Ash and Saito (2006) found that few motorists complied with a 30 km/h speed limit. Even with police enforcement, the average speed still remained 15 km/h above the posted speed limit of 30 km/h in the school zones. In China, the effectiveness of the speed limit sign has not been studied systematically and the feedback information from the Beijing Traffic Management Bureau showed that the effect of speed limits should be improved. Therefore, the speed limit sign is also selected as the focus of this research. According to the *Road Traffic Signs and Markings Manual*, the school zone speed limit is 30 km/h in urban areas. In addition, according to the practical application of traffic control devices in primary school zones and the *Road Traffic Signs and Markings Manual* in China, the placement of the six kinds of signs and markings is shown in Fig. 1.

As shown in Table 2, a total of 17 design alternatives are identified for evaluation in terms of speed control effects. Design Alternative 1 has no school zone signs and markings and is used as the control group. Design Alternative 2 with the School Crossing Ahead Informational Sign and the School Crossing Ahead Warning Assembly is regarded as the existing condition, because these two signs are used in most school zones according to our field investigation. Design Alternatives 3–17 are developed by adding different school zone signs or markings to the existing condition. Specifically, compared with WA and FBWA, WA is one part of FBWA; therefore, WA and FBWA could not appear in one design alternative at the same time. The placement of the six signs and markings is in the sequence shown in Fig. 1.

**Table 2:** The 17 design alternatives of signing and markings in school zones

Design alternative no.	Control group	Existing condition		Flashing Beacon and School Crossing Ahead Warning Assembly	Speed limit sign	Reduce Speed and School Crossing Warning Assembly	School Crossing Ahead Marking
		School Crossing Ahead Informational Sign	School Crossing Ahead Warning Assembly				
1	√						
2		√	√				
3		√		√			
4		√	√		√		
5		√	√			√	
6		√	√				√
7		√		√		√	
8		√		√			√
9		√		√		√	√
10		√	√		√	√	
11		√	√		√		√
12		√	√		√	√	√
13		√	√			√	√
14		√		√	√		
15		√		√	√	√	
16		√		√	√		√
17		√		√	√	√	√

### Scenario

More than 20 primary schools located adjacent to urban streets in Beijing were investigated. According to the street and traffic conditions, and the conflicts between vehicles and pedestrians, researchers summarized the characteristics of the 20 primary schools and developed a classification system. Most schools belong to one of the following two types:

Type I Primary School:

- Located on a major street;
- Four lanes of travel in each direction including one lane for non-motorized traffic;
- Lane width is 3.5–3.75 m;
- Divided by a guardrail median barrier or a grass median strip;
- The school zone is about 500 meters long and the distance from the beginning of a school zone to the school gate is about 300 m;
- The posted speed limit is 60 km/h;
- Traffic volume is about 800 vehicles/direction/h when the school zone is not active;
- Pedestrian traffic is controlled by pedestrian crossing signals.

Type II Primary School:

- Located on a minor street;
- One lane in each direction;
- Lane width is 3–3.5 m;
- Undivided;
- The school zone is about 300 m long and the distance from the beginning of a school zone to the school gate is about 150 m;
- The posted speed limit is 50 km/h;
- Traffic volume is about 250 vehicles/direction/h when the school zone is not active;
- No pedestrian crossing signals.

For each type of school, researchers chose one representative primary school as the object of the study. Then the field scenes for the two representative schools were reproduced in a virtual simulator, according to the actual roadway design features. These were reconstructed for the driving simulator using three-dimensional software and introduced into the simulator scene. The simulator scenes were constructed to represent real scenes as closely as possible.

**Fig. 2:**
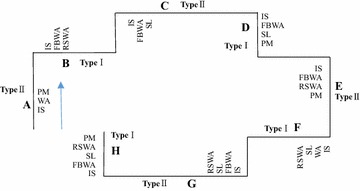
The experimental route for Scenario 1. *WA* School Crossing Ahead Warning Assembly, *IS* School Crossing Ahead Informational Sign, *SL* speed limit sign, *RSWA* Reduce Speed and School Crossing Warning Assembly, *PM* School Crossing Ahead Pavement Markings, *FBWA* Flashing Beacon and School Crossing Ahead Warning Assembly

To eliminate the influence of subjects’ familiarity with the surroundings in the experiment, researchers designed 17 alternatives for the Type I School and 17 alternatives for the Type II School; the two adjacent schools were separated by an 800-m roadway segment. In order to avoid the effects of fatigue caused by driving a long distance, the 34 alternatives for the two representative schools were randomly grouped into five scenarios (see Table 3); the average length of the first four scenarios was about 12.8 km, and the length of the last scenario was about 3.2 km. The route for Scenario 1 is shown in Fig. 2. When subjects drove through the scenario, they proceeded from A to H. The routes for the other scenarios are similar to Scenario 1.

**Table 3:** The five scenarios for the two representative schools in the driving simulator

	Type II	Type I	Type II	Type I	Type II	Type I	Type II	Type I
Scenario 1	WA & IS & School Crossing Ahead Pavement Markings	IS & Flashing Beacon and School Crossing Ahead Warning Assembly & Reduce Speed and School Crossing Warning Assembly	IS & Flashing Beacon and School Crossing Ahead Warning Assembly & speed limit sign	IS & Flashing Beacon and School Crossing Ahead Warning Assembly & speed limit sign & School Crossing Ahead Pavement Markings	IS & Flashing Beacon and School Crossing Ahead Warning Assembly & Reduce Speed and School Crossing Warning Assembly & School Crossing Ahead Pavement Markings	WA & IS & speed limit sign & Reduce Speed and School Crossing Warning Assembly	IS & Flashing Beacon and School Crossing Ahead Warning Assembly & speed limit sign & Reduce Speed and School Crossing Warning Assembly	IS & Flashing Beacon and School Crossing Ahead Warning Assembly & speed limit sign & Reduce Speed and School Crossing Warning Assembly & School Crossing Ahead Pavement Markings
Scenario 2	IS & Flashing Beacon and School Crossing Ahead Warning Assembly & School Crossing Ahead Pavement Markings	IS & Flashing Beacon and School Crossing Ahead Warning Assembly & speed limit sign	Existing Condition (WA&IS)	WA & IS & School Crossing Ahead Pavement Markings	WA & IS & Reduce Speed and School Crossing Warning Assembly & School Crossing Ahead Pavement Markings	IS & Flashing Beacon and School Crossing Ahead Warning Assembly & speed limit sign & Reduce Speed and School Crossing Warning Assembly	IS & Flashing Beacon and School Crossing Ahead Warning Assembly	IS & Flashing Beacon and School Crossing Ahead Warning Assembly & Reduce Speed and School Crossing Warning Assembly & School Crossing Ahead Pavement Markings
Scenario 3	WA & IS & speed limit sign & School Crossing Ahead Pavement Markings	WA & IS & speed limit sign	IS & Flashing Beacon and School Crossing Ahead Warning Assembly & Reduce Speed and School Crossing Warning Assembly	Flashing Beacon and School Crossing Ahead Warning Assembly & School Crossing Ahead Pavement Markings	WA & IS & speed limit sign & Reduce Speed and School Crossing Warning Assembly & School Crossing Ahead Pavement Markings	WA & IS & Reduce Speed and School Crossing Warning Assembly	WA & IS & speed limit sign & Reduce Speed and School Crossing Warning Assembly	WA & IS & Reduce Speed and School Crossing Warning Assembly & School Crossing Ahead Pavement Markings
Scenario 4	IS & Flashing Beacon and School Crossing Ahead Warning Assembly & speed limit sign & School Crossing Ahead Pavement Markings	Existing Condition (WA&IS)	WA & IS & speed limit sign	WA & IS & speed limit sign & School Crossing Ahead Pavement Markings	IS & Flashing Beacon and School Crossing Ahead Warning Assembly & speed limit sign & Reduce Speed and School Crossing Warning Assembly & School Crossing Ahead Pavement Markings	IS & Flashing Beacon and School Crossing Ahead Warning Assembly	WA & IS & Reduce Speed and School Crossing Warning Assembly	WA & IS & speed limit sign & Reduce Speed and School Crossing Warning Assembly & School Crossing Ahead Pavement Markings
Scenario 5	Control	Control						

*WA* School Crossing Ahead Warning Assembly, *IS* School Crossing Ahead Informational Sign

### Subjects

Thirty subjects, including four females, participated in this driving simulation experiment. The male to female ratio of Chinese drivers is about 4:1 (Cui 2014). All were licensed drivers with an average age of 31 (21–57 years, SD = 9.6) and had at least 2 years of driving experience (average = 7.96, SD = 7.17).

### Experiment design and procedure

The fixed-base driving simulator at the Beijing University of Technology consists of a real car, computers, and video and audio equipment. The road scenario is projected onto three large screens, providing a 130° field of view. The simulator is capable of recording the driver’s inputs (e.g., braking, accelerating) 30 times/s. The driving simulator provides a safe and controlled environment to explore how and why drivers react in certain driving situations. The simulated environment allows researchers to inexpensively recreate many different scenarios and test multiple variations of a specific scenario.

The driving simulator experiment included five parts: (1) experimental instructions; (2) a practice drive in the driving simulator; (3) a demographic questionnaire; (4) driving through the five scenarios with random orders, such as 1, 2, 5, 3, 4 or 2, 1, 3, 4, 5; and (5) a post-drive questionnaire.

All subjects were given the following instructions before driving: “You are going to participate in a driving simulator experiment. While driving, please maintain a comfortable and safe speed in accordance with road conditions, and please obey traffic laws and regulations. Please stay in the right lane only. (According to the results of field investigation, more than 77 % of drivers travel in the middle lane when there are three lanes in one direction.) In the event of a car crash during the experiment, please start after the crash location once the system is rebooted.”

The practice drive in the simulator lasted about 10 min. During the practice drive, subjects drove through any one of the five scenarios to familiarize themselves with the simulator’s steering and braking dynamics.

In order to test drivers’ speed choice in different scenarios, no other vehicles were in the same lane as the test car when the subjects drove through the scenarios, but there were vehicles in adjacent and oncoming lanes. Except for signs and markings, everything was identical for the same type of school. The overall experimental driving scenarios were divided into two big blocks according to the length of the scenarios in order to minimize the difference between the lengths of the two blocks, and a long break between these two blocks was arranged. For example, for the order of 1, 2, 5, 3, and 4, Scenarios 1 and 2 belonged to one block, and Scenarios 5, 3, and 4 were in another block. There was a 15-min break between the two blocks to eliminate the effects of fatigue caused by driving for a long period of time. Scenario 5 did not have any school zone signs or markings, serving as a transition between scenarios with various school zone signs and markings. All incidents (e.g., car stalling, crashes, driving off the road, and so on) were recorded for the whole course to simplify data analysis in the future.

After driving in the simulator, subjects should fill out a post-drive questionnaire to evaluate the driving simulator’s ability to mimic real-life conditions, including scenarios, vehicle operations and drivers’ feelings.

### Analysis and results

#### Dependent variables

The 17 design alternatives with speed control devices were defined as the independent variables. Researchers chose four indicators, which were closely related to speed, as the dependent variables. The four dependent variables were listed in Table 4.

**Table 4:** Summary of dependent variables

Variables	Description
Average speed (km/h)	It reflects the overall effectiveness of the design alternative
The relative speed difference	It indicate the degree of the speed change
The standard deviation (SD) of acceleration	It is used to evaluate speed as an indirect evaluation indicator, which depicts the stability of speed reduction through the school zone
The 85th percentile speed (km/h) of all subjects	It is a speed at or below which 85 % of drivers choose to drive, and highly correlated with the speed limit in this area

The data for all the dependent variables were collected between the point of first braking and the point at the school gate. The segment between these two points was defined as the influence area of the design alternative. The first braking point is the location at which the design alternative starts to influence the driver’s speed choice. The data collected at the school gate would help examine the final effectiveness of the design alternative.

Data for the two types of schools were analyzed separately. An analysis of variance with repeated measures (rANOVA) was used to examine the effects and significant differences of different design alternatives on the average speed, relative speed difference and standard deviation (SD) of acceleration. The 85th percentile speed was only a descriptive statistics indicator that served to evaluate the effects of the design alternatives; because it is similar to the average speed, this variable was not included in the rANOVA.

#### Average speed

The effects of the different design alternatives on the average speed were examined using the method of rANOVA.

For the Type I primary school, the results of the rANOVA revealed a significant main effect of different design alternatives on the average speed [F_(16,464)_ = 2.442; *p* = 0.027]. For the 17 design alternatives, a lower average speed meant as better effect. In order to distinguish the differences among them, a Student–Newman–Keuls test (S–N–K test) was employed to categorize the 17 design alternatives. The design alternatives FBWA&PM, FBWA&RSWA&PM, FBWA&SL&PM, RSWA&PM, SL&RSWA&PM, FBWA&SL&RSWA, FBWA&SL&RSWA&PM, FBWA, SL&RSWA, RSWA, FBWA&RSWA, PM, SL&PM, and FBWA&SL were categorized into the same group. Among this group there were no significant differences. The average speed of these design alternatives was lower; specifically, these design alternatives had a better effect on speed reduction.

The same method was also performed and similar results were found for the Type II primary school. There was a significant main effect of the different design alternatives on the average speed [F_(16,464)_ = 6.758; *p* ≤ 0.001]. And the design alternatives PM and FBWA&SL were categorized into the better group.

**Fig. 3:**
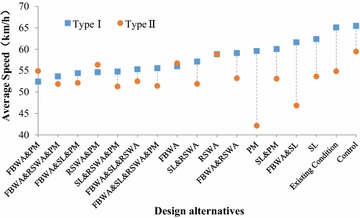
Average speed of each design alterative by school type. *WA* School Crossing Ahead Warning Assembly, *IS* School Crossing Ahead Informational Sign, *SL* speed limit sign, *RSWA* Reduce Speed and School Crossing Warning Assembly, *PM* School Crossing Ahead Pavement Markings, *FBWA* Flashing Beacon and School Crossing Ahead Warning Assembly

Figure 3 presents the comparison between average speeds of the Type I school and the Type II school. The average speeds for the same design alternatives varied greatly by school type. For 13 out of 17 design alternatives, the average speed of the Type I school was higher than the Type II school because the road in Type I school zone had a higher posted speed limit than in the Type II school zone. Obviously, the speed of the design alternative with all signs and markings is not the lowest. The average speed of the control group was the highest because this group had no school zone signs and markings. For the Type I primary school, the minimum speed appeared in the design alternative with FBWA&PM while the design alternative with PM was used for the Type II primary school.

### Relative speed difference

Similar to average speed, the rANOVA and S–N–K test were used to examine the effects of different design alternatives on the relative speed difference, and then to grouping.

Predictably, the researchers found that there were significant differences for the different design alternatives for the Type I primary school [F_(16,464)_ = 8.278, *p* ≤ 0.001] and the Type II primary school [F_(16,464)_ = 7.541; *p* ≤ 0.001]. The design alternatives SL&PM, SL&RSWA&PM, RSWA&PM, FBWA&PM, FBWA&SL&PM, FBWA&SL&RSWA&PM, and FBWA&RSWA&PM were in the group that demonstrated a better effect for the Type I primary school, and SL&RSWA&PM, FBWA&SL&RSWA&PM, PM, FBWA&SL&PM, SL, FBWA&RSWA&PM, SL&PM, SL&RSWA, and RSWA&PM were the better ones of the Type II primary school.

Summarizing the above, according to the analysis results of average speed and relative speed difference, we obtained the optimal design alternatives as presented in Table 5. Design alternatives with a “√” belong to the better group as explained above, and a “*” indicates that there is a significant difference among the 17 design alternatives for this type of school. According to the grouping results, the researchers chose the design alternatives whose average speeds were lower and whose relative speed differences were larger as the objects for further examination. Specifically, if one design alternative belongs to the better group for the two dependent variables simultaneously, the researchers will choose it for further study. Obviously, the comprehensive analysis of the two evaluation indicators showed that the seven design alternatives SL&PM, FBWA&SL&RSWA&PM, SL&RSWA, RSWA&PM, FBWA&SL&PM, FBWA&RSWA&PM, and FBWA&PM are the better ones for Type I school. For the Type II school, the relatively best design alternative is the one with School Crossing Ahead Pavement Markings.

**Table 5:**
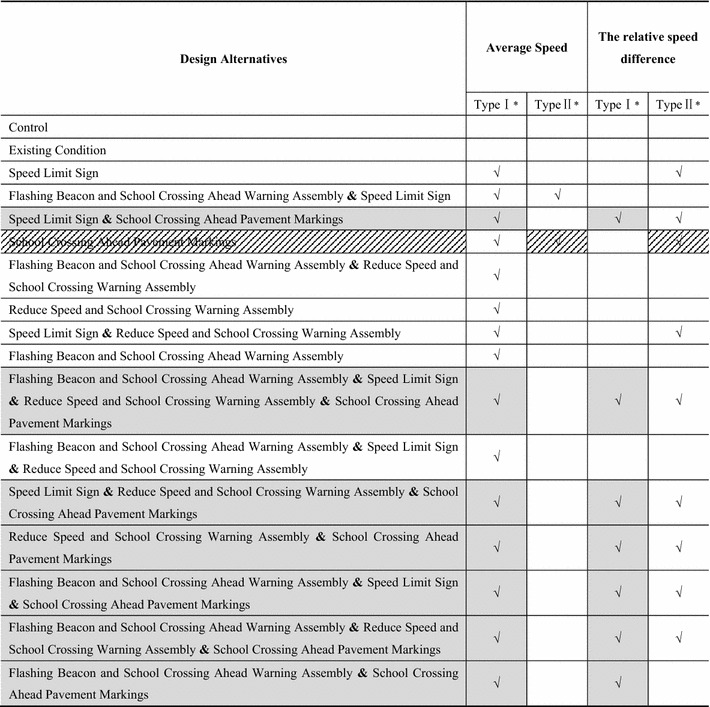
The summary of the better group for different independent variables

### The SD of acceleration

In addition to the two dependent variables associated with speed described above, we took SD of acceleration to reflect the stability of drivers’ speed reduction resulting from the school zone signs and markings.

Similarly, the effect of different design alternatives on the SD of the acceleration was examined using rANOVA.

**Fig. 4:**
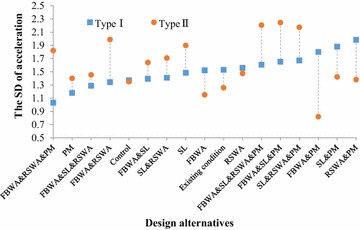
The SD of acceleration of each design alternative for the two types of schools. *WA* School Crossing Ahead Warning Assembly, *IS* School Crossing Ahead Informational Sign, *SL* speed limit sign, *RSWA* Reduce Speed and School Crossing Warning Assembly, *PM* School Crossing Ahead Pavement Markings, *FBWA* Flashing Beacon and School Crossing Ahead Warning Assembly

As expected, no significant differences were found for the design alternatives for the Type I school [F_(16,464)_ = 1.454, *p* = 0.204]. Thus, the 17 design alternatives belong to one group. According to the results of the overall effect of the average speed and the relative speed difference, there were seven design alternatives belonging to the better group: SL&PM, SL&RSWA&PM, RSWA&PM, FBWA&PM, FBWA&SL&PM, FBWA&SL&RSWA&PM, and FBWA&RSWA&PM. As shown in Fig. 4, although there was no significant difference among the seven design alternatives, the SD of acceleration of the design alternative with FBWA&RSWA&PM was the lowest. In other words, when driving through the school zone with FBWA&RSWA&PM, the fluctuation of speed reaches the lowest level. Stated another way, the design alternative with FBWA&RSWA&PM is better than the other six design alternatives.

In addition, for the Type II school, a significant difference was found for the 17 design alternatives on the SD of the acceleration [F_(16,464)_ = 2.353, *p* = 0.038]. An S–N–K test was also used to categorize the 17 design alternatives into different groups. The better group contains FBWA&PM, FBWA, the existing condition, the control group, RSWA&PM, PM, SL&PM, FBWA&SL&RSWA, RSWA, FBWA&SL, SL&RSWA, FBWA&RSWA&PM, SL, and FBWA&RSWA. Notably, the design alternative with the School Crossing Ahead Pavement Markings was included in the better group. This further illustrates that the design alternative with the School Crossing Ahead Pavement Markings was the relatively best one for the Type II school.

As shown in Fig. 4, the control group had a similar effect on the SD of the acceleration for the two types of schools. For the Type II school, the SD of acceleration for more than half of the design alternatives was higher than for the Type I school.

### The 85th percentile speed

The 85th percentile speed was used to evaluate the effects of design alternatives and verify drivers’ speed choice.

**Fig. 5:**
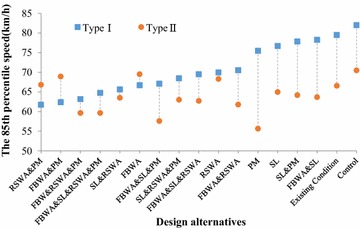
The 85th percentile speed of each design alternative by school type. *WA* School Crossing Ahead Warning Assembly, *IS* School Crossing Ahead Informational Sign, *SL* speed limit sign, *RSWA* Reduce Speed and School Crossing Warning Assembly, *PM* School Crossing Ahead Pavement Markings, *FBWA* Flashing Beacon and School Crossing Ahead Warning Assembly

As shown in Fig. 5, the 85th percentile speed of the design alternative with the Flashing Beacon and the School Crossing Ahead Warning Assembly, the Reduce Speed and School Crossing Warning Assembly, and School Crossing Ahead Pavement Markings for the Type I primary school was not the lowest, but it was lower than most other design alternatives. And from the combined evaluation of the four dependent variables, this design alternative is the relatively optimal one for the Type I primary school. In addition, for the Type II primary school, the 85th percentile speed was the lowest for the design alternative with the School Crossing Ahead Pavement Markings. This further strengthens the conclusion that the design alternative with the School Crossing Ahead Pavement Markings is the relatively best one.

## Discussion

This study examined the effects of 17 traffic control device design alternatives on speed choices in two types of school zones, and the average speed, relative speed difference, SD of acceleration and 85th percentile speed were analyzed. Then the optimal traffic control device design alternatives to reduce speed for the two types of schools were obtained.

In this paper, the researchers first discussed relatively optimal alternatives of different types of schools; as expected, the different representative schools had different relatively optimal traffic control device designs. Roadway and traffic conditions differed for the two types of schools, including the number of lanes and the presence of a median. In order to verify the differences between the two representative schools, rANOVA was also used to analyze the average speed in the field for the two types of schools. We found that there was a significant difference between them [F_(1,29)_ = 6.70, *p* = 0.015]. This finding further strengthened the conclusion that the classification of the two representative schools was correct. This difference between the two representative schools might lead to different relatively optimal traffic control device design alternatives for each type of school. Moreover, previous research studies also found that the success of the school zone speed limits seemed to depend on the traffic and road characteristics (Lee et al. 2006; Tay 2009; Kattan et al. 2011; McCoy et al. 1990). Therefore, the types of schools should be considered before installing traffic control devices in school zones.

Second, while driving speed is definitely the predominant measure for consideration around schools, the selection of appropriate traffic control devices should also consider other factors, such as visibility, legibility, and comprehension. All of these signs and markings are explicitly defined in China’s *Road Traffic Signs and Markings Manual* (2009). Since a thorough understanding of traffic signs and markings is one of the prerequisites for obtaining a driver’s license, we assumed that all drivers are familiar with the signs and markings. Consequently, we did not evaluate drivers’ cognitive ability. Certainly, if possible, the legibility of signs and markings and drivers’ cognitive ability should be studied in further research.

Third, when using rANOVA to examine the effects of different design alternatives on the SD of acceleration, there was a significant difference for the Type II primary school but not for the Type I primary school. This might be associated with the roadway conditions for the two representative schools. The roadway and traffic conditions are better-designed for the Type I school, which helps increase driving confidence. Consequently, it reduces drivers’ dependency on signs and markings. Drivers tended to drive in the curbside lane for Type II school, but they drove in the middle lane for the Type I school; drivers possessed a greater range of visibility in the Type I school than in the Type II school. In brief, the effect of signs and markings are affected by the roadway, traffic conditions, and distance perception. These findings were consistent with the study by Kattan et al. (2011), which found that wider roadways with higher design standards would give drivers the impression of a safer road with a higher speed limit. Further details will be studied in future research.

Furthermore, the S–N–K test was employed to categorize the 17 design alternatives into different groups so as to allow the identification of a better group; from these, we chose the relatively best design alternative. But some design alternatives belonging to both the high-performance group and to the low-performance group were moved to the better group. Doing so would help avoid neglecting any one effective alternative at the greatest extent when evaluating the effectiveness of alternatives in the next step.

Finally, for the two types of schools, the School Crossing Ahead Pavement Markings were an essential part of the relatively best design alternative. Perhaps one simple reason is that these markings are newly emerging devices and drivers are unfamiliar with the markings, so they attract more attention of drivers in engineering practice. Besides, drivers feel a bump while traveling over these pavement markings, and the red color of these markings may also influence drivers’ visual cognitions; therefore, both could make drivers decelerate in school zones. Less obvious but more interesting, although the speed limit sign has a regulatory effect, it doesn’t appear in the relatively best design alternative for the two representative schools. One possible reason may be due to the fact that the penalty for speeding is not heavy enough in China, so speeding is not uncommon; this needs to be further evaluated.

**Fig. 6:**
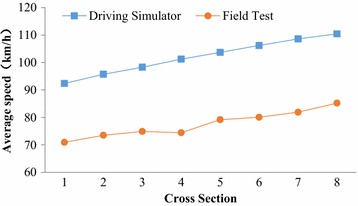
Average speed in straight section

Although our results have not yet been all verified in the field, the performance of the driving simulator at the Beijing University of Technology has been well calibrated and validated in a series of previous studies. About 200 post-drive subjective questionnaires were collected from different experiments using the same driving simulator. The evaluation items of the questionnaire included the accelerator, brake, speed perception and other items. The ratings ranged from 0 (representing “not at all similar to the real world”) to 10 (representing “extremely similar to the real world”); 7.7 and 8.1 represent the ratings of speed perception and scenario, and the ratings of other items were all >7.5 (Xu 2012; Li et al. 2013; Ding et al. 2015). Therefore, the results of the questionnaires support the notion that this driving simulator experiment realistically represents real-world conditions. In addition, Ding et al. (2014) had also validated the travel speeds from the driving simulator using the ground truth speed on a real road. The results showed that the variation trend of these two speeds was consistent(r = 0.990, *p* ≤ 0.05), and the average speed in the driving simulator was about 24 km/h higher than the ground truth speed collected in the field (shown in Fig. 6). Therefore, the average speed of each design alternative with a speed limit sign was higher than the posted speed limit of 30 km/h, as described in Fig. 3. Moreover, the validation results showed that, the driving simulator could be used to conduct research about speed effectiveness. In fact, in China, drivers’ compliance with posted speed limit signs should be carefully discussed; in particular, the speed limit sign in school zone will be the focus of our future research.

**Fig. 7:**
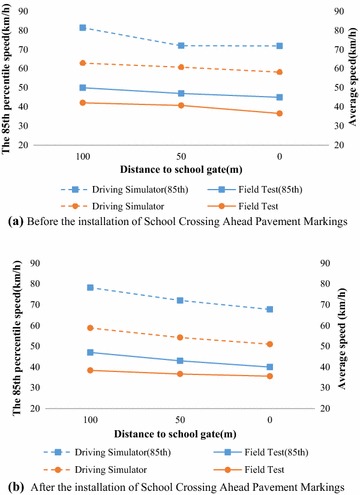
Average speeds and the 85th percentile speeds in field study and simulator experiment, before and after School Crossing Ahead Pavement Markings were paved. **a** Before the installation of School Crossing Ahead Pavement Markings, **b** after the installation of School Crossing Ahead Pavement Markings

Apart from the validation work mentioned above, driving simulator was further validated in this school zone research. Although the objective of this paper is to observe and compare the various effects of different traffic control devices and alternatives, there are not so many alternatives existed in actual. In Beijing, School Crossing Ahead Pavement Markings are widely used. Therefore, we initially compare the average speed related to Type I School in filed observation and driving simulator study, as well as 85th percentile speed. Figure 7a described the average speeds and the 85th percentile speeds in field study and simulator experiment, before School Crossing Ahead Pavement Markings were paved, while Fig. 7b illustrated the results after the installation of School Crossing Ahead Pavement Markings. Statistical results of correlation analysis verified that the variation trend of both speed indicators was consistent in field study and simulator experiment, with and without School Crossing Ahead Pavement Markings. Further validation and calibration will be proceeded.

## Conclusion

This study examined the effectiveness of the 17 design alternatives for reducing speed in each representative school and determined whether the relatively best design alternative was different for the two representative schools.

This study included the following findings:

- Perhaps the most significant contribution of this study was to understand the differences between the two representative schools. Different roadway and traffic conditions led to variations between the relatively best design alternatives. When installing signs and markings in school zones, different design alternatives should be deployed in accordance with different types of schools.
- For the Type I school, the relatively optimal design alternative to control speed was the one with the Flashing Beacon and School Crossing Ahead Warning Assembly, the Reduce Speed and School Crossing Warning Assembly, and the School Crossing Ahead Pavement Markings.
- For the Type II school, the relatively optimal design alternative to control speed was the one with the School Crossing Ahead Pavement Markings.

In brief, the findings demonstrated that when installing signs and markings in school zones, the type of school should be considered because different types of schools require different signs and markings.

### Future research

In this research, the speed of each subject was measured in a dynamic simulated environment, and there were a few differences between the real world and the dynamic simulated environment. In future research, we could validate these findings by comparing the speed in the real world before and after installations of the relatively best design alternative. In addition, we did not study the effect of each sign or marking, but used design alternatives consisting of different signs and markings. A further and more thorough study could be done to classify the signs and markings and determine which signs and markings should be deployed for different types of schools.

## Abbreviations

WA: School Crossing Ahead Warning Assembly
IS: School Crossing Ahead Informational Sign
SL: speed limit sign
RSWA: Reduce Speed and School Crossing Warning Assembly
PM: School Crossing Ahead Pavement Markings
FBWA: Flashing Beacon and School Crossing Ahead Warning Assembly
